# Investigating Inclusive Leadership and Pro-Social Rule Breaking in Hospitality Industry: Important Role of Psychological Safety and Leadership Identification

**DOI:** 10.3390/ijerph19148291

**Published:** 2022-07-07

**Authors:** Sajid Rahman Khattak, Muhammad Zada, Muhammad Nouman, Shams Ur Rahman, Muhammad Fayaz, Rezwan Ullah, Guido Salazar-Sepúlveda, Alejandro Vega-Muñoz, Nicolás Contreras-Barraza

**Affiliations:** 1 Institute of Business and Management Sciences, The University of Agriculture, Peshawar 25130, Pakistan; mnouman@aup.edu.pk (M.N.); shamssayad@aup.edu.pk (S.U.R.); mfayaz@aup.edu.pk (M.F.); 2 Business School, Henan University, Kaifeng 475000, China; 3 School of Management and Economics, Beijing Institute of Technology, Beijing 100081, China; rezwanullah1990@yahoo.com; 4 Departamento de Ingeniería Industrial, Facultad de Ingeniería, Universidad Católica de la Santísima Concepción, Concepción 4090541, Chile; gsalazar@ucsc.cl; 5 Public Policy Observatory, Universidad Autónoma de Chile, Santiago 7500912, Chile; alejandro.vega@uautonoma.cl; 6 Facultad de Economía y Negocios, Universidad Andres Bello, Viña del Mar 2531015, Chile; nicolas.contreras@unab.cl

**Keywords:** inclusive leadership, leadership identification, psychological safety, pro-social rule breaking, hospitality industry

## Abstract

This study aims to empirically examine the mediating effects of psychological safety and leadership identification on the relationship between inclusive leadership and pro-social rule breaking among hospitality employees. This study analyzes the survey data collected in three waves from 589 employees working in different hotels and restaurants operating in the Northern areas of Khyber Pakhtunkhwa, Pakistan. The scale validity, composite reliability, and hypotheses were assessed through PLS-SEM. The study found that inclusive leadership significantly impacts employees’ pro-social rule-breaking. The study also found that leadership identification and psychological safety partially mediate the relationship between inclusive leadership and pro-social rule-breaking. Hospitality leaders can practice inclusive leadership characteristics because it may significantly enhance employee engagement in pro-social rule-breaking. Through their inclusive features, hospitality leaders can improve employees’ psychological safety and leadership identification, enhancing frontline employees’ pro-social rule-breaking.

## 1. Introduction

It is generally observed that service professions like hoteling and restaurants have a high degree of customer contact. The reputation and even long-run survival of service providers depend on frontline employees because they directly contact the customers [1]. Past literature related to service organizations concluded that frontline employees play a crucial role in the overall service experience [2,3,4]. According to [5], there are three main categories of service experience, i.e., mechanic clues, functional clues, and humanistic clues. These service categories were operationalized by [6] in the hoteling industry-defining mechanic clues as to the environmental components of the hotel or restaurant like lighting, design, and layout; functional clues as the food offered by the hotel and restaurant; and humanistic clues as to the behavior and action of service employees, including the level of enthusiasm, body language, and tone of voice [2]. Noted that a restaurant manager could only directly control humanistic and functional clues. Due to high and frequent interaction with customers, frontline employees can adapt their behavior to the feedback they receive from the customers.

Similarly [7], argued that frontline employees have a better sense of what customers demanded from the organization than the organization itself. However, because of this knowledge and expertise, a frontline employee may face a situation where offering better services may result in violating some existing organizational rules and procedures. Generally, violating corporate practices and policies may result in disciplinary action, and in some cases may even lead to termination from the service. Therefore, frontline employees are in a dilemma about offering better services to the customers because providing such services may be subject to criticism by their leaders.

Like other service organizations, the hotel industry is in a continuous struggle to best fit with the external environment, as the customer’s needs, wants, and tastes change rapidly. Today’s customers prefer quality irrespective of what price they paid for it instead of traditional customers whose priority was to avail of services at low prices. Due to this rapidly evolving behavior of the customers, some organizations have failed to adapt their existing rules to the changing demands of customers. This leads to a situation where the current design and regulations that are meant to improve an organization’s efficiency, rather deter its development [8,9]. In such a scenario, proactive employees may break existing organizational rules to improve organizational efficiency. According to [10], the intentional violation of the organizational rules by the employees, with the intention that such action may benefit the organization, its customers, or other stakeholders, is termed pro-social rule-breaking (PSRB). 

PSRB allows managers to develop a unique system that is conducive to organizational development through the identification of deficiencies in the existing rules and procedures [9,11]. Similarly, colleagues can help each other through PSRB while performing job-related tasks and fulfilling their job demands effectively [10,12]. The major reason behind PSRB is the organization’s inability to update the outdated system that hider the work efficiency [12]. For instance, to save time and to improve efficiency, a hotel employee provides room services by himself instead of waiting for an order to be received from their supervisor. In today’s highly competitive and rapidly changing environment, this kind of behavior is not only gaining the attention of employers but also organizational behavior scholars as well. Because such behavior not only improves organizational efficiency and reputation but also motivates the work vivacity of employees [10]. However, very limited attention has been given to this important organizational behavior concept in management literature. Thus, the effort of the present study is to understand the mechanisms of PSRB and its importance in the hospitality industry. 

Overall, the primary purpose behind PSRB is to provide benefits to the organization. To some extent, the existing rules are broken by a person, and he/she is also willing to face the risk of being blamed. As per the notion of Social Information Processing Theory (SIPT), before their engagement in PSRB, employees usually assess whether their behavior will be criticized because leaders are rule makers and its breaker must expect a greater risk of criticism [13,14,15]. Thus, the leadership style is an important determinant of PSRB. Past studies highlighted the link between different types of leadership and PSRB like ethical leadership and PSRB [15,16], and transformational leadership and PSRB [17]. Nevertheless, past studies except [8] have failed to explore whether inclusive leadership (IL) impacts PSRB. 

When employees perceive that their leaders are inclusive, they engage in more PSRB as they believe that they are not blamed by their leaders [18,19]. Even though, the authors expect a positive link between inclusive leadership and PSRB as suggested by [8]. However, this relationship is not always accurate because employees’ perception of their leaders’ behavior varies over time due to cognitive and psychological factors [20]. Employee engagement in PSRB mainly depends on whether he/she wants to do it” and “dare to do it”. The earlier one is the cognitive or motivational state of an employee to challenge the existing rules and procedures and practice new methods and techniques [21,22]. The latter one, “daring to do it”, is the psychological premise of an individual that he/she is not afraid of being blamed due to his/her behavior because they presume higher psychological safety (PsySaf) [23]. Inclusive leaders can increase their followers’ “wanting to do it” and “Daring to do it” by enhancing their leadership identification and psychological safety [24,25]. Thus, it is assumed that both leadership identification and psychological safety might intervene in the relationship between inclusive leadership and PSRB. That is why this study attempts to bridge this gap by investigating whether leadership identification and psychological safety act as an intervening mechanism between inclusive leadership and the PSRB relationship. 

This study aims to empirically investigate the direct and indirect link between inclusive leadership and PSRB via LI and PsySaf amongst hospitality employees. The present research offers novel theoretical contributions. Though, the motive behind PSRB is to provide benefits to the organization, co-workers, and customers. But before their engagement in PSRB, employees will assess whether their behavior will be criticized because leadership style is an important determinant of PSRB [13,14,15]. Therefore, the authors expect that an inclusive leadership style would play a major role in the PSRB. This is because when employees feel that their leaders are inclusive; their fear of being criticized by their leaders is low which ultimately leads to a higher inclination towards PSRB. However, despite being an important determinant of PSRB, the extant literature has relatively ignored this dimension. Thus, this study is among the pioneering studies that establish a linkage between PSRB and inclusive leadership. 

**Figure 1:**
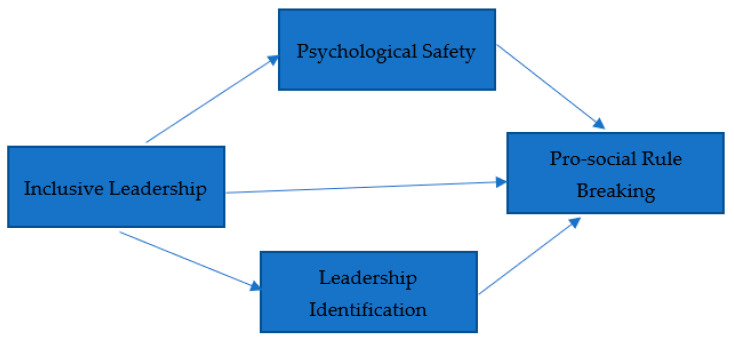
Conceptual Framework of the Present Research.

This study also examines the intervening mechanisms of PsySaf and LI on the relationship between INCL and PSRB. The behavior of the leader and his actions steer the individual psychological changes. When employees have a higher level of PsySaf, their level of “daring to do it” is increased, which ultimately induces them to engage more in PSRB. Individuals with higher PsySaf dare to be involved in risky behaviors and vice versa. A high level of PsySaf is attained through a safe and supportive environment. Thus, the characteristics of inclusive leadership also provide support to employees to achieve a high level of PsySaf [24]. The present study also found a strong positive association between INCL and PsySaf, thus supporting the theoretical foundation provided by [23]. Hence, INCL affects PSRB directly and indirectly via PsySaf. Figure 1 presents the conceptual framework of the study.

## 2. Literature Review and Research Hypotheses

### 2.1. Inclusive Leadership and Pro-Social Rule Breaking

Inclusive leadership is a leadership style where a leader encourages followers’ contributions and actively listens to their views [26]. The followers believe that there is someone who attends and values their voice. While interacting with employees, such leaders express specific dominant characteristics like accessibility, availability, and openness [27]. Among them, accessibility is the leader’s ability to develop a strong relationship with their followers by giving due attention to their needs [28]. Availability is the leader’s ability to timely help their followers when facing difficulties in performing their work. Openness is the leadership quality where leaders motivate their followers to think creatively, bring new ideas, listen to their opinions actively, and involve them in decision-making [29,30]. 

On the other hand, PSRB is the employee’s behaviors where they deliberately break an organization’s rules, policies, and procedures for the more considerable interest of the organization or some of its stakeholders [10]. If an organization cannot update its system, it may enhance employees’ involvement in PSRB [12]. In a society where societal innovations are encouraged or where people think more positively about their organizations or peers, more people will be involved in PSRB because it improves organizational efficiency and enhances the work vivacity of employees [31].

Consistent with social information processing theory (SIPT), people’s attitudes may get affected by the information they receive from the social environment. Individuals interpret the social information in different ways which ultimately determine their subsequent behaviors. Though, individuals can’t receive all social information and pay attention to all of them. They only interpret specific information and practice such behaviors that are acceptable to their organization [13]. Ref. [32] argued that the leader is the prominent source of social information for employees in the work setting. As employees involve in PSRB, they assess the possible consequences of such actions. Employees’ involvement in PSRB is high when they believe that such behavior meets their leader’s expectations, and their involvement is low when they feel that their behavior will be criticized [12]. Ref. [33] suggested that employees’ worries concerning the outcomes of their conduct will dissipate when they perceive that their leaders have inclusive characteristics. Thus, it is proposed that:

**H1.**  *Inclusive leadership is positively related to hoteling employees’ PSRB*.

### 2.2. The Role of Psychological Safety

Psychological safety is the psychological state where employees perceive that they are safe concerning their career, status, and self-image. When employees’ concern regarding the negative consequences of their behavior is low, they openly express themselves in an organization (Hu et al., 2018). Based on SIP theory [13], an employee engaging in PSRB deliberately violates organization rules and procedures, and he/she is at risk of being punished. Thus, employees need to assess the environment before engaging in PSRB. Ref. [24] argued that only those employees would involve in PSRB who have a high level of psychological safety and firmly believe that their leaders will not punish them for their actions. Thus, psychological safety may be responsible for employees “daring to do it” wish. 

However, the higher level of psychological safety of an employee depends on a supportive and inclusive environment, and under the umbrella of inclusive leadership, employees may find such an environment [23]. As previously reported, inclusive leaders motivate their followers to come up with new ideas and provide help when employees face difficulties in performing job-related tasks [34]. Thus, working under such leadership allows employees to perceive that breaking rules will not be criticized; instead, they expect that their leaders will show support and help. Such perception reduces the risk of adverse consequences of being involved in PSRB and enhances their psychological safety and makes them dare to be involved in PSRB. Thus, it is proposed that:

**H2a.**  *Psychological safety is significantly related to hotel employees’ PSRB*.

**H2b.**  *Employees’ psychological safety fully mediates the relationship between inclusive leadership and hoteling employees’ PSRB*.

### 2.3. The Role of Leadership Identification (LI)

Ref. [35] Argued that a state where an employee positions himself/herself in an organization based on his/her relationship with the leaders is termed leadership identification. On the other hand [36], argued that leadership identification is shaped when employees’ perception of the leader is unified into their self-concept. Employees consider their leader a reference point when they have a high level of LI, which further inspires them to practice values like their leader and self-concept. Additionally, they wish to change their existing cognitive concepts according to their leader [37]. However, as per leader-member exchange (LMX) theory, leadership effectiveness is not only dependent upon what the leader does but largely on the exchange relationship between employees and leaders [23]. Employees’ leadership identification is largely influenced by the information they acquire and the behaviors of their leaders [14]. Thus, we expect that good LMX not only improves followers’ positive perception of INCL but also increases LI, which may ultimately enhance PSRB [23,30]. 

Inclusive leadership characteristics enhance employees’ “dare to do it” and motivate employees “want to do it”. Ref. [38] Argued that the psychological makeup behind “wanting to do it” comes from employees’ internal motivation and cognition. Employees’ identification with their leaders may influence the association between inclusive leadership and PSRB. Leader’s inclusive characteristics such as accessibility, availability, and openness impress the followers and increase the trust and loyalty level they have in their leader [39]. Ref. [40] Suggested that such leaders instill their values in their followers (identification) and make them able to achieve what they expect through positive behaviors. Hence, when the leaders demand new ways to solve problems or to improve the existing process, employees having higher LI and conceptual internalization may take necessary actions for the benefit of the organization or its stakeholders, even if such act of an employee breaks some rules or procedures of an organization. Thus, it is proposed that:

**H3a.**  *Leadership identification is significantly related to hotel employees’ PSRB*.

**H3b.**  *The relationship between inclusive leadership and hoteling employees’ PSRB is fully mediated by leadership identification.*

## 3. Materials and Methods

### 3.1. Sample and Procedure

Data was collected through survey data collected from 589 employees working in different hotels and restaurants operating in the Northern areas of Khyber Pakhtunkhwa. The study contacted employees working in different hotels and restaurants located in Northern areas of Khyber Pakhtunkhwa province of Pakistan. The HR managers of 80 three stars and four stars hotels were contacted to fix the schedule of the survey. After receiving consent from 65 HR managers, employees were contacted to voluntarily participate in the study with the agreement that their responses will be kept confidential and will only be used for research purposes. To minimize common method bias [41,42,43,44,45], (a bias that occurs when the data of independent and dependent variables are collected from the same person/source, in the same measurement context using the same item context and similar item characteristics) data was collected in three waves with two months break in each wave. Researchers, e.g., refs. [41,44,45] suggest that the CMV effect could be minimized by collecting the data at different intervals. The researchers personally visit each hotel for data collection. The data was collected from all levels of employees. The total time of data collection was 7 months. To collect the data about inclusive leadership, the study distributed 1500 questionnaires among the hotel staff at time point 1. The study received 860 responses out of 1500 targeted samples with a response rate of 57.33%. Data regarding psychological safety and leadership identification was collected at time point 2 (two months after time point 1) by contacting those 860 respondents who participated in the survey at time point 1. The study assigned a unique identification number to every participant making us able to trace those 860 respondents who actively participated in the survey at time point 1. The study received 622 responses from the participants at time point 2 with a response rate of 72.32%. Finally, at time point 3, two months after time point 2, data was collected from the employees regarding their PSRB. The study target 622 employees (those who participated in the previous two surveys) to participate in the final survey at time point 3. The study received 589 valid responses from the target respondents with a response rate of 96.14%. The overall response rate of the current study was 39.26%. 

### 3.2. Measurement

Inclusive Leadership (INCL) was measured through a nine items scale developed by [23]. This scale measures inclusive leadership in three basic dimensions, i.e., openness, accessibility, and availability. The sample item is “My leader is ready to listen to my request”. All items were measured with a five-point Likert scale where 1 represents strongly disagree, and 5 represents strongly agree. A past study found good reliability of this scale, i.e., 0.93 (e.g., Wang and Shi, 2020). This study also found an excellent CR value, i.e., 0.96. 

To measure Psychological Safety (PsySaf) the present research adopts a five items scale developed by [46]. All items were rated on a five-point Likert scale where 1 represented strongly disagree and 5 means strongly agree. The CR value of this scale is 0.93. 

The scale developed by [47] was used to measure Leadership Identification (LI). This scale has six items and was measured on a five-point Likert scale. The sample item is “Praising my leader feels like I am being praised”. For this scale, this study received an excellent CR value, i.e., 0.93. 

A scale developed by [12] on Pro-social Rule Breaking (PSRB)was used in this study. This scale has thirteen items that measure three significant dimensions, i.e., customer assistance, co-worker assistance, and efficiency. However, the current study used PSRB as a composite variable; thus, all thirteen items represent PSRB only. The sample item is “To provide better customer services; I violate organizational rules”. All items were assessed on a five-point Likert scale where 1 = strongly disagree, and 5 = strongly agree. This study received an excellent CR value for this scale, i.e., 0.96.

## 4. Results

Data were analyzed using partial least square structural equation modeling PLS-SEM. In PLS-SEM, there are two models, i.e., the measurement model and the structural model. The details of both are reported below. 

### 4.1. Measurement Model

**Figure 2:**
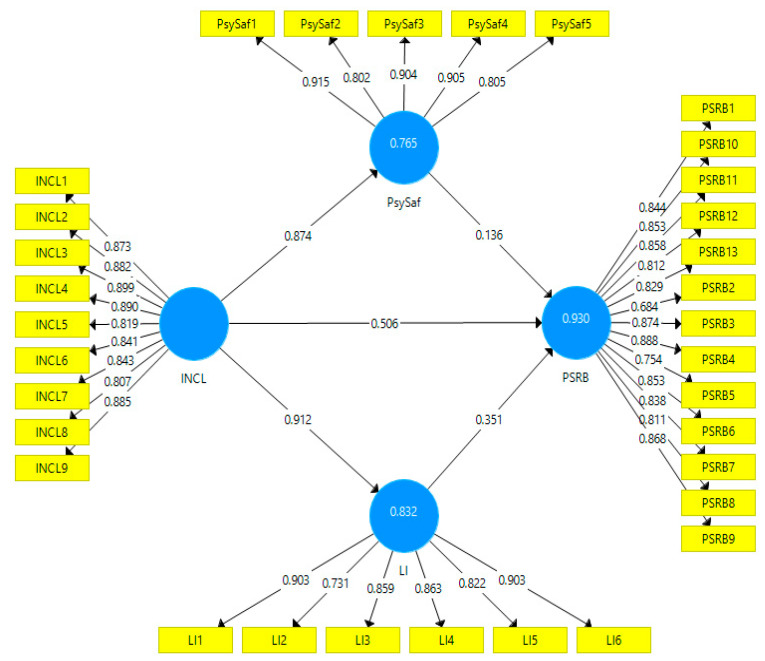
Measurement Model.

The relationship between the constructs and indicators was tested using a measurement model (See Figure 2). The measurement model requires that the scale achieve good reliability and validity. The scales used in the current study have good reliability as all values of Cronbach’s alpha and composite reliability (CRs) are well above the threshold value of 0.70 (see Table 1). This study assured convergent validity through average variance extracted (AVE) as the values of AVE for all variables are higher than the recommended value of 0.50 (see Table 1). Discriminant validity was assured through factor loading, HTMT ratio, and Fornell-Larcker criteria. The values of all items of all variables are well above the recommended value of 0.50; thus, all items were retained in the model (see Table 1). Through the Fornell-Larcker criteria, this study also established discriminant validity as the values reported in the upper diagonal of the table are the Square-roots of AVE, and it should be greater than the inter-constructs correlation (see Table 2). HTMT ratio shows that the correlation among all constructs is below 0.85, thus ensuring discriminant validity (see Table 3).

### 4.2. Structural Model

**Figure 3:**
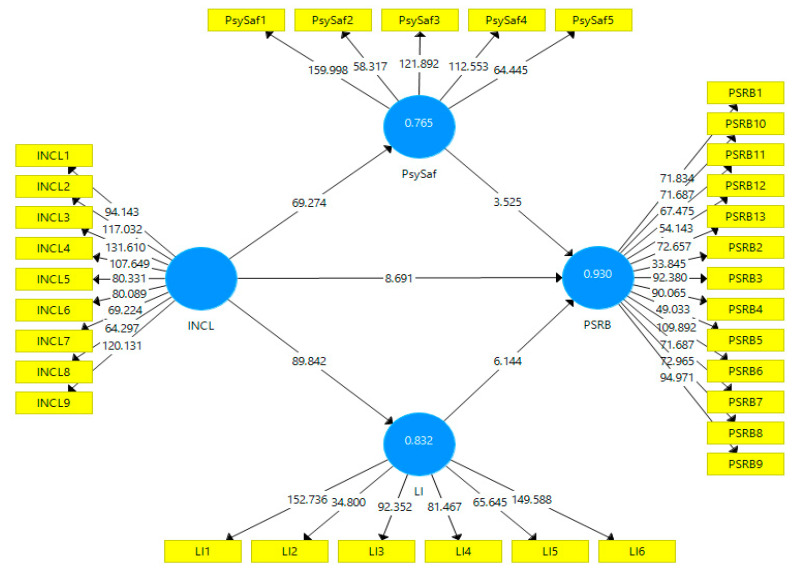
Structural Model.

The structural model (See Figure 3) tests the hypothesized framework of the study. This study assessed the structural model based on path significance, Q^2^, and R^2^. The strength of every structural path determines the goodness of the model. R^2^ shows the strength of each structural path, and its value should be equal to or more than 0.10 [48]. The values of R^2^ for all three paths are well above the suggested value; thus, the predictive capability is established. The endogenous constructs’ predictive relevancy was assessed through Q^2^, and its value should be greater than 0. Here the values of Q^2^ are more than 0 which shows the significant predictive relevance of the constructs (see Table 4). Furthermore, keeping in view the recommendation of [49], this study also tests the model fit through SRMR. The value of SRMR was 0.068, indicating the excellent model fit as the value falls under the threshold value of 0.10.

#### Hypotheses Testing and Research Findings

**H_1_.**  *Inclusive leadership is positively related to hoteling employees’ PSRB*.

The study tests the hypotheses to assess the significance level with 5000 bootstrapping samples at 95% confidence. H_1_ shows whether INCL has a significant impact on PSRB. The findings suggest that INCL has significant impact on PSRB (β = 0.502, t = 9.48, *p* < 0.05). When the t value is equal to or above the standard range of ±1.96 and the *p*-value is less than 0.05 then we can say that the relationship between IDV and DV is significant. This means that: when leaders have high INCL characteristics, it increases employees’ involvement in PSRB. Hence, this study received support for H_1_. H_1a_ shows whether INCL has a significant impact on PsySaf. 

The findings suggest that INCL has significant impact on PsySaf (β = 0.874, t = 69.75, *p* < 0.05). This means that: employees feel psychologically safe if leaders have INCL characteristics. Hence, the study received support for H_1a_. H_1b_ shows whether PsySaf has a significant impact on PSRB. The findings suggest that PsySaf has significant impact on PSRB (β = 0.151, t = 4.31, *p* < 0.05). This suggests that when employees feel that they are psychologically safe, their engagement in PSRB will be high. Hence, the study received support for H_1b_. 

Similarly, H_1c_ shows whether INCL has a significant impact on LI. The findings suggest that INCL has significant impact on LI (β = 0.912, t = 89.52, *p* < 0.05). This means that: when leaders have high INCL characteristics, employee positions himself/herself in an organization based on his/her relationship with the leaders will be high. Hence, the study received support for H_1c_. H_1d_ shows whether LI has a significant impact on PSRB. The findings suggest that LI has significant impact on PSRB (β = 0.341, t = 6.84, *p* < 0.05). This means that: when employees’ LI is high, their engagement in PSRB will be high they position himself/herself in an organization based on his/her relationship with the leaders. Hence, the study received support for H_1d_ (see Table 4).

**H_2_.**  *Employees’ psychological safety fully mediates the relationship between inclusive leadership and hoteling employees’ PSRB*.

Based on the findings of the current research, psychological safety partially mediates the relationship between INCL and employees’ PSRB i.e., PsySaf (β = 0.132, t = 4.23, *p* < 0.05). This means that: the relationship between INCL and PSRB is passed through PsySaf. Simply, INCL effect PsySaf and then PsySaf effect PSRB (see Table 5). Thus, H_2_ of the current study is supported.

**H_3_.**  *The relationship between inclusive leadership and hoteling employees’ PSRB is fully mediated by leadership identification*.

The study found that leadership identification partially mediates the relationship between INCL and employees PSRB, i.e., (β = 0.310, t = 6.80, *p* < 0.05). It means the relationship of INCL with PSRB is passed through LI. Simply, INCL effect LI and then LI affect PSRB. Thus, H_3_ is supported. 

### 4.3. Mediation Analysis

This study applied mediation analysis to test whether PsySaf and LI play an intervening role in the relationship between INCL and PSRB. The results show that (see Table 5) both study mediators i.e., PsySaf (β = 0.132, t = 4.23, *p* < 0.05) and LI (β = 0.310, t = 6.80, *p* < 0.05) partially mediates the link between INCL and PSRB. 

## 5. Conclusions

The rapid changes in the external environment compel organizations to change at the same pace. The existing rules and complex procedures hinder the organizations from changing to what the external environment demanded. This may not only limit the organization to development but also restrict employees from taking initiative. Such situations may create room for proactive employees to engage in PSRB with the intention that such initiatives may favor their colleagues, customers, and organizations. PSRB can not only help to sustain employees’ eagerness but also help in inducing system reforms. 

Today’s organizations can use pro-social rule-breaking as an internal mechanism to pace their development with environmental changes. This study empirically examines the relationship between INCL and PSRB among hotel and restaurant employees working in different hotels in the Northern areas of Khyber Pakhtunkhwa, Pakistan. In addition, the study also tests whether PsySaf and LI intervene in this relationship? This study was based on three major objectives. 

1. The first objective was to examine the relationship between INCL and PSRB among hotel employees. The study found that INCL is positively and significantly related to PSRB. It means that when leaders actively practice INCL characteristics in a hotel setting, the engagement of hotel employees in PSRB tends to increase. Thus, the first objective of the study is achieved. The findings of the current research are consistent with [8,27,29,30]. 

2. The second research objective was to investigate the mediating role of PsySaf on the relationship between INCL and PSRB. This study found that PsySaf partially mediates the relationship between INCL and PSRB. It means that INCL effect PsySaf and then PsySaf effect PSRB. Simply, the relationship between INCL and PSRB is passed through PsySaf. Thus, the second objective of the study is achieved. The findings of the present research are in line with [23,24,34]. 

3. The third objective of the present study was to investigate the mediating role of LI on the relationship between INCL and PSRB among hotel employees. This study found that LI partially mediates the relationship between INCL and PSRB. It means that INCL effect LI and then PsySaf effect PSRB. Simply, the relationship between INCL and PSRB is passed through LI. Thus, the study received support for its third objective. The findings of the present research are in line with [39,40]. 

This study contributes theoretically in novel ways. Though, the motive behind PSRB is to provide benefits to the organization, co-workers, and customers. But before their engagement in PSRB, employees assess whether their behavior will be criticized because leaders are rule makers, and one’s must expect a greater risk of criticism [13,14,15]. However, when employees feel that their leaders are inclusive; their fear of being criticized by their leaders is low which ultimately leads to a higher inclination towards PSRB. Thus, inclusive leadership plays an important role in inducing PSRB. However, despite being an important determinant of PSRB, the extant literature has relatively ignored this dimension. Thus, this study is amongst the pioneering studies that establish a linkage between PSRB and inclusive leadership. 

This study also examined the intervening mechanisms of PsySaf and LI on the relationship between INCL and PSRB. As rightly noted by [50], the behavior of the leader and what he/she does is accountable for individual psychological changes. When employees have a higher level of PsySaf, their level of “daring to do it” increases, which ultimately provokes them to engage more in PSRB. Thus, individuals with higher PsySaf dare to be involved in risky behaviors and vice versa. A high level of PsySaf is attained through a safe and supportive environment. Thus, the characteristics of inclusive leadership provide support to employees to achieve a high level of PsySaf [24]. The present study also found a strong positive association between INCL and PsySaf, thus supporting the theoretical foundation provided by [23]. Hence, INCL affects PSRB directly and indirectly via PsySaf.

On the other hand, individual involvement in PSRB depends on inner safety and whether they “want to do it” as [51] noted that individuals’ internal thinking and motivation could also determine their behavior. Thus, when engaging in PSRB, they will use their rational concepts to judge whether they violate the existing rules for the sake of organizational benefits or some of its stakeholders as inclusive leaders motivate their followers to offer new methods for developing the organization. For organizational development, followers come up with new ways and try to implement them (wanting to do it), even if such methods may violate some established rules [21]. The study results also support this statement as the current study found that LI intervenes in the relationship between INCL and PSRB.

### 5.1. Managerial Implications

This study offers certain implications for managers. Based on the current study findings, it is found that inclusive leadership traits positively affect hotel employees’ engagement in PSRB. Thus, through their characteristics, inclusive leaders would encourage their employees to practice PSRB for the organization’s welfare, motivate them to come up with new methods, and help them to think creatively. All these practices encourage employees to do something special for the organization even if the existing rules are violated. Top management should focus on how to develop their leaders’ inclusive characteristics. Furthermore, top management suggested that, instead of blaming their employees for rules breaking, they could try to understand the underlying intentions to know why particular employees are engaged in PSRB. By doing so, top management should provide relevant training and workshops to make their leaders able to understand the characteristics of inclusive leadership. 

PSRB plays a crucial role in organizational development, and it may be the only way for organizations to respond timely to the rapidly changing external environment. Thus, it is suggested that hospitality managers to some extent may encourage their employees to engage in PSRB. HR managers can improve their employees’ PSRB by creating a culture where employees feel that their contributions are acknowledged instead of being criticized. HR managers should guide their employees to know when and where to break the existing rules of the organization. 

In addition, as this study found that PsySaf and LI are proportionally accountable for employees’ engagement in PSRB, hospitality leaders can improve their followers PsySaf and LI through their actions and positive communication. By doing so, they can enhance their followers’ “wanting to do it” and daring to do it” because the psychological makeup behind “wanting to do it” comes from employees’ internal motivation and cognition. Hospitality leaders can practice various effective communication strategies and allow their followers to bring new ideas for organization development as the inherent characteristics of inclusive leaders like encouraging innovation and creativity, actively listening to their employees, and involving them in decision-making would encourage their followers to engage in PSRB. Such practices may improve their followers’ level of PsySaf and LI and enhance their engagement in PSRB which can enable organizations to adjust to the pace of the external environment. 

### 5.2. Limitations and Future Directions

Since no research is free of limitations. There are some potential limitations of this research as well that may provide directions to future researchers. First, the study used PSRB as a composite variable ignoring its dimensions, i.e., organizational efficiency, customers support, and co-workers support because past studies [10] argued that employees involve in PSRB to improve organizational efficiency instead of co-worker’s support and customers’ support. Hence, this study fails to reveal for what reason (i.e., organizational efficiency, co-workers support, customers support) do employees involved in PSRB. Future researchers can eliminate this limitation by selecting all three dimensions of PSRB and coming up with more robust findings. Second, to minimize the CMV effect, the study chooses a time-lag research design by collecting the data at three points in time. However, the generalizability of the findings is still limited. Thus, for the generalizability of findings, future researchers can select a longitudinal research design to investigate this relationship. Lastly, this study was conducted in an abnormal condition, i.e., the peak period of COVID-19, and could not capture an accurate picture of the respondents (i.e., hospitality employees) involvement in PSRB in normal conditions. Thus, it would be better to replicate the study model in normal organizational settings.

## Figures and Tables

**Table 1:** Factor Loading, Alpha, CR, and AVE.

Factor	A	Alpha	CR	AVE
Inclusive Leadership		0.95	0.96	0.74
INCL1	0.873			
INCL2	0.882			
INCL3	0.899			
INCL4	0.890			
INCL5	0.819			
INCL6	0.841			
INCL7	0.843			
INCL8	0.807			
INCL9	0.855			
Psychological Safety		0.91	0.93	0.75
PsySaf1	0.915			
Psysaf2	0.802			
PsySaf3	0.904			
PsySaf4	0.905			
PsySaf5	0.805			
Leadership Identification		0.92	0.93	0.72
LI1	0.903			
LI2	0.731			
LI3	0.859			
LI4	0.863			
LI5	0.821			
LI6	0.902			
Pro-social Rule Breaking		0.96	0.96	0.68
PSRB1	0.847			
PSRB2	0.688			
PSRB3	0.875			
PSRB4	0.889			
PSRB5	0.754			
PSRB6	0.850			
PSRB7	0.839			
PSRB8	0.808			
PSRB9	0.870			
PSRB10	0.850			
PSRB11	0.857			
PSRB12	0.810			
PSRB13	0.830			

**Table 2:** Fornell-Larcker Criteria.

	INCL	LI	PSRB	PsySaf
INCL	0.860			
LI	0.812	0.849		
PSRB	0.824	0.823	0.830	
PsySaf	0.847	0.749	0.730	0.868

**Table 3:** HTMT Criteria.

	INCL	LI	PSRB	PsySaf
INCL				
LI	0.812			
PSRB	0.824	0.823		
PsySaf	0.847	0.749	0.730	

**Table 4:** Path Coefficients.

	Original Sample (O)	(STDEV)	T Statistics	*p* Values	2.50%	97.50%
INCL → PSRB	0.502	0.053	9.487	0.000	0.396	0.594
INCL → PsySaf	0.874	0.013	69.758	0.000	0.847	0.895
PsySaf → PSRB	0.151	0.035	4.314	0.000	0.081	0.218
INCL → LI	0.912	0.01	89.529	0.000	0.890	0.929
LI → PSRB	0.341	0.05	6.845	0.000	0.244	0.431
	**R^2^**	**Q^2^**				
LI	0.832	0.595				
PSRB	0.931	0.634				
PsySaf	0.765	0.570				

**Table 5:** Mediating Effects.

	Total Effect	t	Sig	Direct Effect	t	Sig	Indirect Effect	Effect	t	Sig
INCL-PSRB	0.944	171.49	0.000	0.502	9.48	0.000	INCL-PsySaf-PSRB	0.132	4.23	0.000
							INCL-LI-PSRB	0.310	6.80	0.000

## Data Availability

By request to the authors of correspondence.
